# Identification of VPS13C as a Galectin-12-Binding Protein That Regulates Galectin-12 Protein Stability and Adipogenesis

**DOI:** 10.1371/journal.pone.0153534

**Published:** 2016-04-13

**Authors:** Ri-Yao Yang, Huiting Xue, Lan Yu, Antonio Velayos-Baeza, Anthony P. Monaco, Fu-Tong Liu

**Affiliations:** 1 Department of Dermatology, School of Medicine, University of California-Davis, Sacramento, California, 95817, United States of America; 2 School of Life Sciences, Northeast Normal University, Changchun, 130024, People’s Republic of China; 3 Wellcome Trust Centre for Human Genetics, OX3 7BN, Oxford, United Kingdom; 4 Institute of Biomedical Sciences, Academia Sinica, Nankang, Taipei, 115, Taiwan; University of Liverpool, UNITED KINGDOM

## Abstract

Galectin-12, a member of the galectin family of β-galactoside-binding animal lectins, is preferentially expressed in adipocytes and required for adipocyte differentiation *in vitro*. This protein was recently found to regulate lipolysis, whole body adiposity, and glucose homeostasis *in vivo*. Here we identify VPS13C, a member of the VPS13 family of vacuolar protein sorting-associated proteins highly conserved throughout eukaryotic evolution, as a major galectin-12-binding protein. VPS13C is upregulated during adipocyte differentiation, and is required for galectin-12 protein stability. Knockdown of *Vps13c* markedly reduces the steady-state levels of galectin-12 by promoting its degradation through primarily the lysosomal pathway, and impairs adipocyte differentiation. Our studies also suggest that VPS13C may have a broader role in protein quality control. The regulation of galectin-12 stability by VPS13C could potentially be exploited for therapeutic intervention of obesity and related metabolic diseases.

## Introduction

Galectins are a family of β-galactoside-binding animal lectins with one or two conserved carbohydrate-recognition domains (CRDs) [1,2]. These proteins are mainly cytosolic as their genes do not encode any recognizable signal peptides and are therefore excluded from the classical ER/Golgi secretory pathway. However, some galectins can be found outside the cell in various amounts, depending on the proliferation/activation states of the cells, and are believed to be secreted via nonclassical secretory pathways [3,4]. Extracellular galectins are usually thought to interacts with N-acetyllactosamine (LacNAc)-containing glycans of glycoproteins and glycolipids on the cell surface to modulate endocytosis and cell signaling pertinent to cell activation, proliferation, differentiation, and death [5–8]. Galectins also act inside the cell where complex glycans are mostly absent, and are thought to function through protein-protein interactions to regulate apoptosis, pre-RNA splicing, and energy metabolism [9,10]. Some intracellular galectins monitor the integrity of endosomes and lysosomes that contain invading bacteria by binding to host glycans exposed on damaged vacuoles [11]. Extracellular and intracellular functions of galectins imply that these proteins are involved in physiological and pathological conditions, such as in the immune response and cancer [1,2].

Galectin-12 has two CRDs separated by a linker sequence and is preferentially expressed in adipocytes [12,13], whose dysfunctions links obesity to insulin resistance and type 2 diabetes [14]. We have shown that galectin-12 is required for adipogenic signaling and adipocyte differentiation *in vitro [15]*, and have recently found that the protein is associated with adipocyte lipid droplets to regulate lipolysis [16,17]. Ablation of galectin-12 reduces adiposity and alleviates glucose intolerance and insulin resistance associated with weight gain [17]. To understand the mechanism of action of galectin-12, it is important to identify its upstream regulators and downstream mediators. We therefore set out to identify proteins that interact with galectin-12. Here we show that VPS13C, a member of the VPS13 family of proteins (VPS13A, B, C, and D), is a galectin-12-binding protein that is associated with lipid droplets and lysosomes to regulate galectin-12 stability in adipocytes. VPS13C is upregulated during adipocyte differentiation and is required for galectin-12 protein expression and adipogenesis. The functions of mammalian VPS13 proteins remain mostly elusive and this study provides the first experimental functional data for VPS13C.

## Materials and Methods

### Materials

Polyclonal galectin-12 antibodies were generated in galectin-12 knockout mice [17]. Mouse anti-FLAG M2 antibody and Anti-FLAG M2-agarose were purchased from Sigma. They were used for Western blotting and immunoprecipitation of Flag-tagged proteins. For immunofluorescence, rabbit anti-Flag M2 antibody (Cell Signaling Technology) was used. Mouse anti-LAMP1 and anti-Myc tag antibodies were from Millipore and Syd Labs, respectively. MitoTracker Deep Red, rabbit anti-LAMP1 and anti-Myc tag antibodies were purchased from Cell Signaling Technology. Rabbit anti-perilipin-1 was from Affinity Bioreagents. Rabbit anti-calnexin was purchased from Stressgen. The following cell lines were purchased from ATCC: mouse fibroblast cell line 3T3-L1 (ATCC CL-173), human embryonic kidney cell line 293T (ATCC CRL-11268), and the human cervical carcinoma cell line HeLa (ATCC CCL-2). They were cultured following standard conditions in DMEM/10% FBS at 5% CO_2_, 37°C.

### Generation of VPS13C antibodies

A cDNA fragment encoding positions 1582–1882 of human VPS13C isoform 2A (UniProtKB Q709C8-1) (84% identities, 92% positives with mouse VPS13C protein Q8BX70-1, positions 1580–1879) was amplified with primers C3F (5’-AAGTTCTGTTTCAGGGCCCGATCGCTGTCAAAGCTGTATCC-3’) and C3R (5’-ATGGTCTAGAAAGCTTTACAAAATTTTCATTAAAACTGTCAAG-3’), using Deep Vent DNA polymerase (NEB) and a full-length *VPS13C* cDNA plasmid as a template, and sub-cloned into pOPINF vector as previously described [18] to obtain plasmid pF-C3.Rosetta(DE3) LysS*E*. *Coli* bacteria transformed with pF-C3 were grown on Overnight Express^TM^ Instant TB medium (Novagen), and processed as previously described [19]. The over-expressed N-terminally His-tagged VPS13C fragment, mostly present in the insoluble fraction, was extracted overnight at 4°C with solubilization buffer (6 M urea, 50 mM Tris pH7.8, 300 mM NaCl, 30 mM Imidazole, 1 mM DTT) and purified with Ni-NTA agarose (QIAGEN). Solubilized purified protein in elution buffer (6 M urea, 50 mM Tris pH7.8, 300 mM Imidazole) was used for rabbit immunization to raise polyclonal antiserum C-F3-R1 (obtained from Eurogentec Ltd).

### Generation of DNA constructs

DNA inserts encoding full-length galectin-12 protein or individual CRDs with three copies of FLAG tag (3xFLAG) were generated by PCR using the high-fidelity DNA polymerase PicoMaxx (Stratagene), and cloned into the pSC-A-amp/kan vector using the StrataClone PCR Cloning Kit (Stratagene). After confirmation by sequencing, the inserts were excised and cloned into the pMSCVpuro retroviral vector (Clontech). The resultant constructs encode 3xFlag-tagged versions of LC3 (3F-LC3), full-length galectin-12 (3F-G12, aa1-314), C-CRD-deleted galectin-12 (3F-G12dC, aa1-189), and N-CRD-deleted galectin-12 (3F-G12dN, aa162-314). For mammalian over-expression of a C-terminally myc-His-tagged human VPS13C protein, several overlapping *VPS13C* cDNA fragments amplified by RT-PCR [20] and cloned into pGEM-T (Promega) were combined to obtain a full-length cDNA insert without stop codon, corresponding to variant 1A (GeneBank AJ608770, positions 1 to 11204), that was sub-cloned between *Hin*dIII (blunt) and *Xba*I sites in pcDNA4-TO-mycHis (Invitrogen) to generate plasmid pcD13C6. This insert contains SNPs rs3784634, rs2303405, rs11629598, rs12907567, rs10851704 and rs765205705, at positions 2866, 3850, 4428, 6909, 8683 and 9968, respectively. We used a retro-lentiviral system for conditional gene knockdown through doxycycline-regulated expression of artificial amiRs [21]. For each gene, we designed oligonucleotides targeting three different regions using Invitrogen’s Block-iT RNAi Designer program (Table 1), and cloned them into the lentiviral vector pMA2867 between the two BfuAI sites, flanked by the 5' and 3' sequences from the mouse miR-155 gene [21]. The insert was confirmed by sequencing before the construct was used to generate lentiviruses.

**Table 1:** RNAi design for specific knockdown of indicated genes with amiRs.

Species	Gene	No.	Region	Sequence
*Escherichia coli*	*LacZ*	1	324–344	TACGGTCAATCCGCCGTTTGT
*Escherichia coli*	*LacZ*	2	496–516	AGTCGTTTGCCGTCTGAATTT
*Escherichia coli*	*LacZ*	3	819–839	GCCTTTCGGCGGTGAAATTAT
*Mus musculus*	*Lgals12*	1	446–466	TATCTGGTGACATCTTGGTAA
*Mus musculus*	*Lgals12*	2	511–531	AGAGAGTATCCAGTTGGATAT
*Mus musculus*	*Lgals12*	3	786–806	GCGATTCTTCGAGGTACTGCT
*Mus musculus*	*Vps13c*	1	847–867	GCACATCTTGGAGCAACTGAA
*Mus musculus*	*Vps13c*	2	1206–1226	CATGGTCATGGAGTAACATAA
*Mus musculus*	*Vps13c*	3	1374–1394	CACAAGTTGAGGTCATTCATT

Three top-scoring target regions in the open reading frame of each gene were selected using Invitrogen’s Block-iT RNAi Designer. Coordinates of the target sequences are relative to the start of the coding region of each gene.

### Generation of retrovirusesand lentiviruses

We produced viruses by packaging viral particles into 293T cells. For retrovirus production, we co-transfected cells with the transfer vector, the packaging plasmid pUMVC, and the envelope plasmid pMD2.G. Lentiviruses were produced similarly, except that pPax2 was used as the packaging plasmid. Transient transfection of 293T cells was performed using FuGENE 6 Transfection Reagent (Promega). Viral supernatants were harvested and pooled 2 and 3 days after transfection.

### Identification of galectin-12-binding proteins

We transduced 3T3-L1 cells by incubating cells with pMSCVpuro-3xFLAGm12 (3xFLAG tagged mouse galectin-12) retroviruses and 8 μg/ml polybrene. Cells were selected one day later in medium containing 1 μg/ml puromycin for 5 days. The whole puromycin-resistant cell population was expanded and then cryopreserved or used for experiments. To identify galectin-12-binding proteins, these cells were induced to differentiate into adipocytes [22]. A total of ∼3 × 10^7^ 3T3-L1 adipocytes transduced with control retrovirus or retrovirus expressing 3xFLAG-galectin-12 were rinsed with PBS and then lyzed in 1 ml of ice-cold buffer containing 50 mM Tris (pH 8), 120 mM NaCl, 0.5% NP-40, and Protease Inhibitor Cocktail (Sigma). After centrifugation, supernatants were incubated with 30 μl anti-FLAG M2 agarose beads (Sigma) at 4°C for 2 h. Beads were then spun down, washed 6 times in lysis buffer, and eluted by boiling 5 min in 30 μl 2x SDS-sample buffer. Proteins in eluted samples were then separated on a SDS-PAGE gradient acrylamide gel (4–15%, Bio-Rad) and visualized by silver staining. Protein bands of interest were excised and submitted to UC Davis Proteomics Core for in-gel protein digestion and identification by LC-MS/MS. Mass spectrometry results were searched using X!tandem and search results were returned using the Scaffold software. Samples from four independent IP experiments were analyzed, either as total immunoprecipitates or isolated gel bands. All came back with highly consistent results.

### Isolation of lipid droplets

Lipid droplets were isolated from 3T3-L1 adipocytes by density gradient centrifugation, as described [17,23].

### Deconvolution immunofluorescence microscopy

Cells were fixed with paraformaldehyde, permeabilized with digitonin, and further processed for immunostaining of cellular proteins with specific primary antibodies and appropriate corresponding fluorescence-labeled secondary antibodies, as described [17]. Where indicated, lipid droplets and nuclei were stained with 1 μg/ml Bodipy 493/503 (Invitrogen) and 1 μg/ml Hoechst 33342 (Invitrogen), respectively. Fluorescent signals were visualized using a BX61 fluorescence microscope (Olympus) and z-plane images at 0.5-μm intervals encompassing the depth of the cell were captured. Flat-field-corrected image stacks were deconvolved using Huygens software (Scientific Volume Imaging). Colocalization analysis was performed using the Coloc 2 plugin of ImageJ.

### Generation of doxycycline-regulated knockdown cell lines

We generated conditional knockdown stable cell lines by sequential transduction. Cells were first transduced with the pMA2641 retrovirus by incubation with viral supernatant and 8 μg/ml polybrene. One day later, cells were switched to fresh medium containing 10 μg/ml Blasticidin S and selected in this medium for 5 days. The whole blasticidin-resistant cell population was then transduced with the pMA2867 lentiviruses targeting *LacZ* (control), *galectin-12*, or *Vps13c* genes, using the same method. After 5 days of selection in medium containing 1 μg/ml puromycin, the surviving cell population was either used for experiments or cryopreserved. To induce gene knockdown, these co-transduced cells were cultured in the presence of 1 μg/ml doxycycline for 3 days, and subjected to gene expression analysis by real-time RT-PCR and immunoblot, as described [17]. Primer pairs used for real-time RT-PCR are listed in Table 2.

**Table 2:** DNA oligos used for Q-PCR.

Species	Gene	No.	Sequence
*Mus musculus*	*Lgals12*	1	AAAGTGACTTAAGACTCTGTCTCCTGG
*Mus musculus*	*Lgals12*	2	CTAAGTAACCTCAACCACCAACCTGCC
*Mus musculus*	*Vps13c*	1	ATCATTCGTCCATATGACAGGCAGGAATCG
*Mus musculus*	*Vps13c*	2	GAAGCCTTGTAGAATCCATGGTCAAGACGG
*Mus musculus*	*PPIA*	1	CTGCACTGCCAAGACTGAATGGCTGGATGG
*Mus musculus*	*PPIA*	2	GGACGCTCTCCTGAGCTACAGAAGGAATGG

### Statistical analysis

Data are presented as means ± standard error (s.e.). Measurements in control and experimental groups were compared by unpaired two-tailed Student’s t-tests using Prism 5 (GraphPad Software, Inc.). Results were considered statistically significant at p < 0.05.

## Results

### Identification of VPS13C as a galectin-12-binding protein

**Fig 1:**
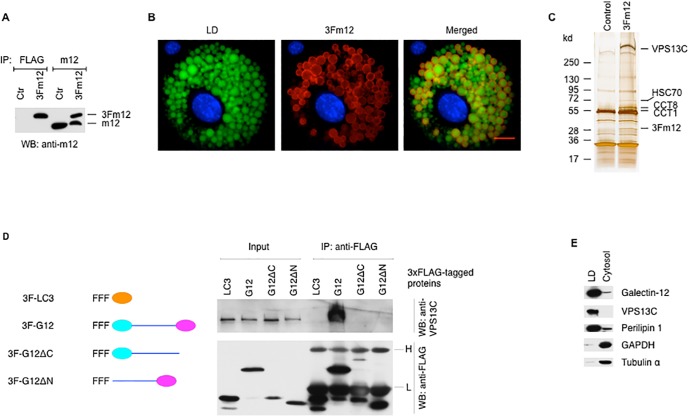
Identification of VPS13C as a galectin-12-binding protein. (**A**) Lysates from 3T3-L1 adipocytes transduced with a control retrovirus (Ctr) or one that expresses 3xFLAG tagged mouse galectin-12 (3Fm12) were immunoprecipitated with either an anti-FLAG or anti-galectin-12 antibody, as indicated. Immunoprecipitates were further immunoblotted with a galectin-12 antibody. (**B**) Immunofluorescence of 3T3-L1 adipocyte expressing 3Fm12 with anti-FLAG antibody (red). Cells were co-stained with Bodipy 493/503 and Hoechst 33342 to reveal lipid droplets (LD, green) and the nucleus (blue), respectively. Scale bar, 10 μm. (**C**) Lysates of control 3T3-L1 adipocytes or those expressing 3Fm12 were immunoprecipitated with anti-FLAG antibody and the immunocomplexes were analyzed by LC-MS/MS. (**D**) Lysates from 293T cells transducedwith 3xFLAG-tagged LC3 (control), full-length galectin-12 or truncated mutants lacking the N- or C-terminal CRD were immunoprecipitated with anti-FLAG M2-agarose beads, and total lysates or immunoprecipitates were analyzed by immunoblotting with anti-VPS13C or anti-FLAG antibodies. H and L denote the heavy and light chains of IgG, respectively. (**E**) Cytosol and lipid droplet fractions of 3T3-L1 adipocytes were immunoblotted with indicated antibodies. Results are representative of three experiments.

We chose a system to stably express 3xFLAG-tagged galectin-12 in adipocytes as the bait, followed by purification of galectin-12-containing protein complexes by immunoprecipitation with an anti-FLAG tag antibody, and then identification of the binding proteins by mass spectrometry (LC-MS/MS). Because endogenous galectin-12 is preferentially expressed in adipocytes, this method subjects the bait protein to a relevant cellular context, increasing the likelihood of identifying bona fide protein interactions. Similar approaches have been successfully used to show that the zinc finger protein PRDM16 (PR domain containing 16) specifies the brown fat lineage from a progenitor by binding to the transcription factors PPARγ [24] and C/EBPβ [25], as well as the transcriptional repressor CtBP [26]. The retrovirally transduced 3xFLAG-tagged galectin-12 protein was not detectable in 3T3-L1 fibroblasts until these cells were induced to differentiate into adipocytes, in which the levels of transduced galectin-12 were comparable to those of endogenous galectin-12 (Fig 1A). This is important as overexpression can cause mislocalization and spurious interaction with other proteins. As expected, the bait protein, like endogenous galectin-12 [17], was localized on adipocyte lipid droplets (Fig 1B). Mass spectrometry analysis of anti-FLAG immunoprecipitate of 3xFlag galectin-12-expressing cell lysates identified several protein bands that were absent from the immunoprecipitate of control lysates. These include the protein chaperons heat shock cognate 71 kDa protein (HSC70) and T-complex protein 1 (TCP-1) subunit α and θ (CCT1 and CCT8) (Fig 1C and Table 3). Of particular interest was VPS13C, a member of the VPS13 family of vacuolar protein sorting proteins that is highly conserved throughout eukaryotic evolution [20,27].

**Table 3:** Galectin-12-binding proteins identified by affinity purification-MS using 3xFLAG-tagged mouse galectin-12 as a bait.

Identified Proteins	Gene symbol	MW (kd)	Unique peptides	Coverage (%)
Vacuolar protein sorting 13 homolog C	*Vps13c*	415	49	17
T-complex protein 1 subunit θ	*Cct8*	60	3	6.6
Heat shock cognate 71 kDa protein	*Hspa8*	71	3	6.2
T-complex protein 1 subunit α	*Cct1*	55	2	4.3

Galectin-12 consists of two carbohydrate-recognition domains connected by a linker sequence [13]. To determine which domain of galectin-12 is involved in its interaction with VPS13C, we expressed 3xFLAG-tagged wildtype full-length galectin-12 or its truncated mutants in HEK293T cells, using 3xFLAG-tagged LC3 as a negative control. Immunoprecipitation with an anti-FLAG tag antibody followed by immunoblot with an anti-VPS13C antibody showed that only 3xFLAG-tagged wildtype full-length galectin-12 could be co-immunoprecipitated with VPS13C (Fig 1D). The results suggest that both domains of galectin-12 are required for interaction with VPS13C. Cellular fractionation experiments showed that both galectin-12 and VPS13C were enriched in adipocyte lipid droplets (Fig 1E).

### Galectin-12 and VPS13C are co-upregulated during adipocyte differentiation and required for adipogenesis

**Fig 2:**
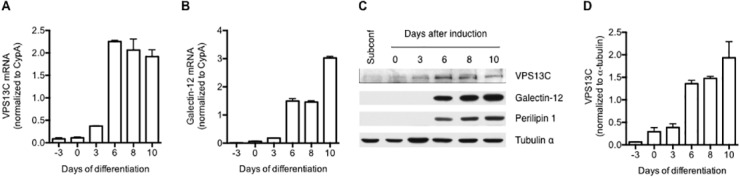
Galectin-12 and VPS13C are co-upregulated during adipocyte differentiation. Expression was assayed by quantitative real-time RT-PCR for mRNA levels (**A and B**) and by immunoblotting for protein levels (**C and D**) in subconfluent 3T3-L1 fibroblasts, or at different time points of adipocyte differentiation. Adipocyte differentiation was induced at day 0, when cells were three days post confluence, following an established adipogenic regimen [22]. Bar graphs present data (means ± s.e.) from three experiments.

Previous studies have shown that human *VPS13* genes are ubiquitously expressed in many tissues and cell types examined; however, their expression in adipocytes has not been investigated [20]. We examined the expression of *Vps13c* during adipocyte differentiation of 3T3-L1 cells induced with an adipogenic cocktail [22] and found that the gene was markedly upregulated after adipocyte differentiation. After 3 days of induction, *Vps13c* mRNA was upregulated 13-fold (relative to subconfluent cells) and its levels plateaued by day 6 (Fig 2A). In comparison, galectin-12 was upregulated following slower kinetics. Although the galectin-12 gene was clearly induced after 3 days, high expression was not detected until 6 days, and it plateaued after 10 days (Fig 2B). These results were further confirmed at the protein level by immunoblotting (Fig 2C and 2D).

**Fig 3:**
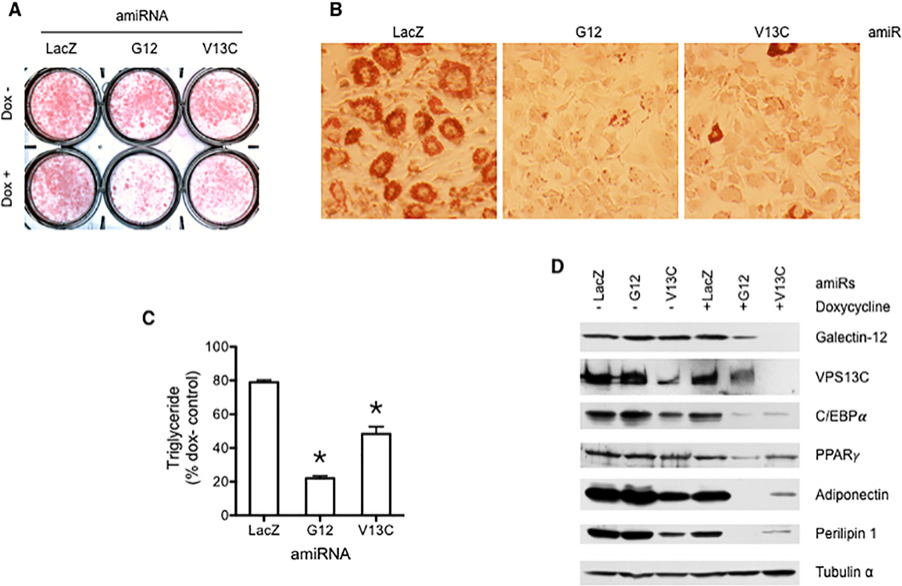
Like galectin-12, VPS13C is required for adipocyte differentiation. 3T3-L1 cells were stably transduced with a doxycycline-inducible conditional knockdown system for *LacZ* (control), galectin-12, or *Vps13c*. Cells were then stimulated to undergo adipocyte differentiation following an established regimen for 10 days, in the continuous absence or presence of doxycycline. Adipocyte differentiation was assayed by Oil-Red-O staining of neutral lipids (**A** and **B**), by quantification of triglycerides with AdipoRed (**C**), and by immunoblotting of indicated adipocyte proteins (**D**). Asterisks denote statistical significance (*P < 0.05). Results are representative of three to four experiments.

Upregulation of VPS13C during adipogenesis suggests that, like galectin-12, this protein could also play a role in adipocyte differentiation. We set out to test this by using a conditional knockdown system [21]. 3T3-L1 fibroblasts were transduced with a retrovirus that expressed the reverse tetracycline-controlled transactivator rtTA-Advanced, and a lentivirus that carries a tetracycline-responsive cassette to express an artificial microRNA (amiR) targeting the gene of interest. After transducing 3T3-L1 cells with the system targeting *LacZ* (control), *galectin-12*, or *Vps13c*, respectively, we induced adipocyte differentiation in the absence or presence of doxycycline, and determined adipocyte differentiation and the expression of *galectin-12* and *Vps13c* genes 10 days after induction (Fig 3). Continued expression of *Vps13c* and *galectin-12* amiRs resulted in nearly complete depletion of VPS13C protein, and partial (76%) depletion of the galectin-12 protein, respectively (Fig 3D). As expected, knockdown of galectin-12 markedly suppressed adipocyte differentiation, as judged by reduced neutral lipid accumulation (Fig 3A–3C) and decreased expression of several adipose genes (Fig 3D). Importantly, knockdown of *Vps13c* also suppressed adipocyte differentiation, but to a lesser extent, as judged by the above parameters (Fig 3).

### VPS13C is required for galectin-12 protein stability in adipocytes

**Fig 4:**
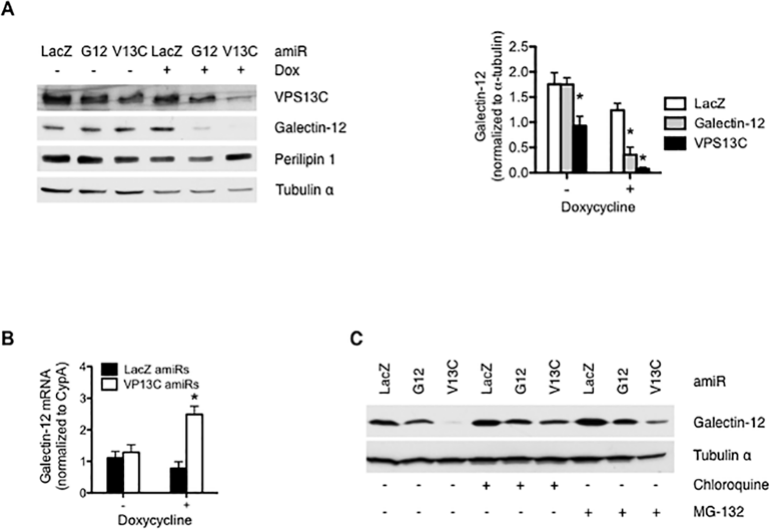
VPS13C is required for galectin-12 protein stability in adipocytes. 3T3-L1 cells were stably transduced with a doxycycline-inducible conditional knockdown system for *LacZ* (control), galectin-12, or *Vps13c*. Cells were then stimulated to undergo adipocyte differentiation for 7 days and then treated with doxycycline for 3 days to induce gene knockdown. Protein levels and galectin-12 mRNA levels were determined by immunoblotting (**A**) and quantitative RT-PCR (**B**), respectively. (**C**) 3T3-L1 adipocytes transduced with the above system were treated with doxycycline for 3 days in the absence or presence of the lysosome inhibitor chloroquine or the proteasome inhibitor MG-132 before immunoblot assay with galectin-12 or tubulin antibodies. Results are representative of three experiments. Asterisks denote statistical significance.

Unexpectedly, we found that *Vps13c* knockdown markedly decreased galectin-12 protein levels (Fig 3D). This is not secondary to suppression of adipocyte differentiation, as *Vps13c* knockdown resulted in a lesser suppression of differentiation but a greater reduction of galectin-12 levels than *galectin-12* knockdown (Fig 3). In support of this, reduced galectin-12 protein levels were found even when *Vps13c* knockdown was induced after adipocyte differentiation (Fig 4A). Decreased galectin-12 protein levels in *Vps13c* knockdown cells were not due to decreased mRNA levels, as *galectin-12* mRNA levels were actually higher in these cells (Fig 4B). The above results suggest that the observed effects at the protein level were most likely due to accelerated galectin-12 degradation. This effect is specific for galectin-12, as the levels of another lipid droplet protein, perilipin-1, were not reduced by *Vps13c* knockdown (Fig 4A).

Most cellular proteins are degraded by one or both of the two major protein degradation pathways: the ubiquitin–proteasome system and the autophagic-lysosomal pathway [28,29]. While the proteasome degrades soluble proteins, its narrow barrel preclude entry of large protein aggregates, which need to be cleared by autophagic-lysosomal pathway [30–32]. To determine which degradation pathway is primarily responsible for the differential degradation of galectin-12 between control and *Vps13c* knockdown cells, we treated cells with specific inhibitors of these pathways and monitored galectin-12 levels. Depletion of VPS13C in the presence of the lysosome inhibitor chloroquine almost completely prevented the decrease in galectin-12 levels, and partial prevention was observed when these cells were treated with the proteasome inhibitor MG-132 (Fig 4C). These results suggest that VPS13C depletion accelerates galectin-12 degradation mainly through the lysosomal pathway.

**Fig 5:**
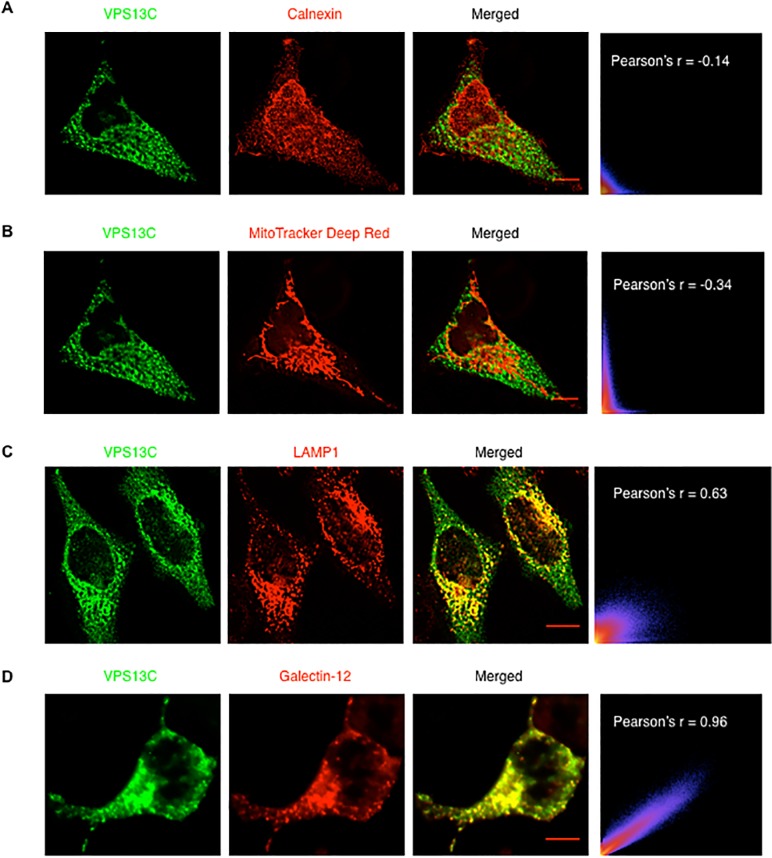
VPS13C colocalizes with lysosomes and galectin-12. (**A** and **B**) HeLa cells transfected with Myc-tagged VPS13C were first stained with MitoTracker Deep Red, fixed, permeabilized and immunostained with mouse anti-Myc tag and rabbit anti-calnexin antibodies. (**C**) HeLa cells transfected with Myc-tagged VPS13C were immunostained with mouse anti-LAMP1 and rabbit anti-Myc antibodies. (**D**) HeLa cells were transduced with a retrovirus encoding 3xFLAG-tagged galectin-12 and transfected with Myc-tagged VPS13C. Cells were then immunostained with mouse anti-FLAG and rabbit anti-Myc antibodies and appropriate fluorescence-labeled secondary antibodies. Deconvolved image stacks were analyzed for colocalization of the red and green signals with the Coloc 2 plugin of ImageJ. Scale bar, 10 μm. Results are representative of three experiments.

As the quality of the available antibodies against VPS13C is not high enough for subcellular localization of the endogenous protein, we determined the localization of transfected Myc-tagged human VPS13C in HeLa cells. No colocalization was found of VPS13C with the ER marker calnexin (Fig 5A) or the mitochondrial marker MitoTracker Deep Red (Fig 5B). We found that, consistent with its regulation of galectin-12 degradation through the autophagy-lysosome pathway, VPS13C colocalized with the lysosome marker LAMP1 (Fig 5C) and transduced galectin-12 (Fig 5D), with Pearson's coefficient of 0.63 and 0.96, respectively.

### VPS13C does not regulate overall autophagy but may have a broader role in protein quality control

**Fig 6:**
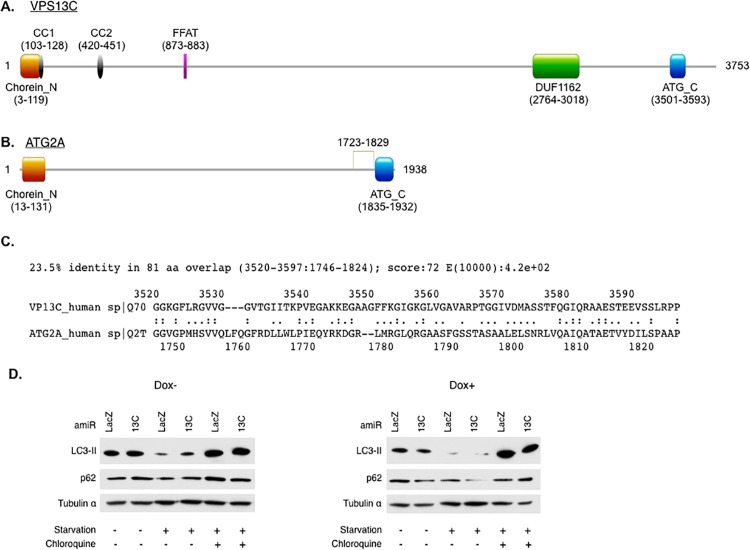
*Vps13c* knockdown does not affect bulk autophagy. Both human VPS13C (**A**) and ATG2 (**B**) carry a Chorein_N and a ATG_C domains. cc, coiled coil. Data from the Pfam protein families database [57]. (**C**) An ATG-C-nearby sequence in ATG2A (1723–1829), essential for autophagy and required for ATG2A localization to both the autophagic membrane and lipid droplets [33], is also conserved in VPS13C. (**D**) 3T3-L1 fibroblasts engineered with a doxycycline-regulated knockdown system for control (LacZ) or *Vps13c* gene were treated for 3 days with or without doxycycline. Cells were then cultured 3 h under basal conditions, or in a medium depleted of amino acid and serum (starvation), in the absence or presence of chloroquine. Cells were lysed and analyzed by immunoblotting for indicated proteins. Results are representative of three experiments.

VPS13C shows similarities in domain architecture to ATG2A, an autophagy-related protein known to regulate autophagy and lipid droplet morphology in mammalian cells [33,34]. Both proteins carry a Chorein_N domain and an ATG_C domain (Fig 6A and 6B) and a sequence critical for ATG2A function is partially conserved in VPS13C (Fig 6C), suggesting that VPS13C could also regulate autophagy. Interestingly, the closely related VPS13 A has been reported to regulate autophagy [35]. We assessed the autophagy flux in *Vps13c* knockdown cells by measuring the dynamics of the autophagosome marker LC3-II and the autophagy substrate p62 in the absence or presence of the lysosome inhibitor chloroquine under basal or autophagy-stimulating (starvation) conditions [36]. No significant differences in LC3-II or p62 levels were found between control and *Vps13c* knockdown cells under these conditions (Fig 6D), suggesting that VPS13C depletion does not affect autophagy. Taken together, the results suggest that galectin-12 is selectively targeted for accelerated autophagic-lysosomal degradation in *Vps13c* knockdown cells, and VPS13C does not regulate the overall autophagy pathway.

**Fig 7:**
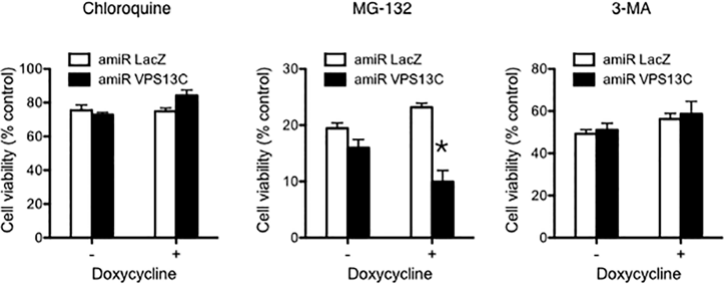
VPS13C knockdown sensitized cells to proteasome inhibition. 3T3-L1 fibroblasts engineered with the doxycycline-induced LacZ or VPS13C knockdown system were treated for three days without or with doxycycline in the presence of chloroquine, MG-132, or 3-MA. Cell viability was then analyzed using MTS assay[58]. Results are representative of three experiments. Asterisks denote statistical significance.

Blockage of the ubiquitin–proteasome system causes accumulation of damaged proteins that are toxic to cells and very often leads to cell death [37]. To test whether VPS13C is generally involved in this system, we treated cells with the proteasome inhibitor MG-132 and found enhanced death of VPS13C-depleted cells (Fig 7). This is not a result of the general sensitivity of *Vps13c* knockdown cells to cell death, as inhibition of autophagosome formation with 3-MA or late autophagy with chloroquine caused comparable cell death in control and *Vps13c* knockdown cells (Fig 7). Instead, the results suggest the involvement of VPS13C in protein degradation by the ubiquitin–proteasome system.

## Discussion

Galectin-12 has an important role in triglyceride metabolism and yet the mechanism is not well understood. Our work identifies VPS13C as a galectin-12-binding protein, and suggests that it could have a major role in protein quality control. We further show that it regulates autophagic-lysosomal degradation of selected proteins, and may also be generally involved in the ubiquitin–proteasome system.

The most interesting finding of this study is that VPS13C is required for galectin-12 stability. VPS13C depletion greatly reduced galectin-12 levels as a result of increased degradation by lysosomes. Decreased galectin-12 protein levels in VPS13C knockdown cells contrast with increased galectin-12 mRNA levels, suggesting an attempt by the cells to compensate for the loss of this protein by upregulating the transcription or by stabilizing the mRNA of this gene. We showed that both CRDs of galectin-12 are required for VPS13C interaction, yet the mechanism of how galectin-12 is targeted by VPS13C is not clear. Galectin-12 has hydrophobic stretches that span both the N- and C-terminal domains [17] and is hard to heterologously express in soluble form in bacterial or mammalian cells, suggesting that it is an aggregation-prone protein. Its preferential degradation by the autophagic-lysosomal pathway is therefore consistent with the fact that protein aggregates are degraded via this pathway, while soluble damaged proteins undergo degradation in the ubiquitin–proteasome system [30–32]. It is also consistent with the observation that VPS13C colocalizes with what seem to be galectin-12 aggregates and lysosomes. Accelerated degradation of galectin-12 through the autophagic-lysosomal pathway is selective in *Vps13c* knockdown cells as overall autophagy does not change in these cells. Galectin-12 levels were partially restored in VPS13C-depleted cells when cells were incubated with MG-132, and these cells were also more sensitive to cell death induced by this proteasome inhibitor, suggesting that VPS13C is generally involved in protein degradation through the ubiquitin–proteasome system.

Besides VPS13C, additional proteins that were identified by the affinity capture-MS method as possible galectin-12-binding proteins are all known chaperones that facilitate protein folding [38], including the CCT1 and CCT8 subunits of the eukaryotic chaperonin TRiC/CCT, as well as HSC70, a constitutively expressed member of the HSP70 family. Inside the cell, VPS13C and galectin-12 colocalize and they are closely associated with lipid droplets and lysosomes. It therefore appears that the task of keeping galectin-12 in an active state may involve the concerted actions of VPS13C with several protein chaperones and organelles. Lipid droplets have been shown to interact with cytosolic inclusion bodies in yeast and facilitate their clearance by producing a sterol-based metabolite [39]. It appears that galectin-12 and lipid droplets may mutually regulate each other. On one hand, galectin-12 is required for lipid droplet formation [15–17]; on the other hand, these organelles may also mediate galectin-12 clearance, through concerted actions with VPS13C and other protein chaperones.

Many human diseases are caused by the dysregulated accumulation of ubiquitinated protein aggregates, including neurodegenerative disorders and some diseases that affect muscles, heart or liver. It is possible that like the p62-interacting adaptor protein ALFY [40,41], VPS13C regulates autophagy of protein aggregates (aggrephagy) but not bulk autophagy, and plays a general role in the homeostasis of aggregation-prone endogenous proteins. This could be a likely function in view of its reported genetic association with Parkinsons disease [42,43] and late onset Alzheimer's disease [44].

In yeast, Vps13 is implicated in vacuolar protein sorting, TGN-endosomal cycling of membrane proteins, and prospore membrane morphogenesis during sporulation, probably through regulating PtdIns(4)P localization and levels [45]. Whether VPS13C has parallel functions in mammals remains to be determined, but high throughput screening has identified several VPS13C-binding Rab GTPases that serve as master regulators of intracellular membrane trafficking, including Rab1A, Rab9A, Rab30, and Rab7 [46,47].

We have previously shown that galectin-12 is a protein preferentially expressed in adipocytes and plays roles in adipocyte differentiation and lipolysis important for the regulation of whole-body adiposity and glucose homeostasis. In this paper, we further identify VPS13C as a galectin-12-binding protein required for galectin-12 protein stability and show that VPS13C depletion also impairs adipogenesis. The fact that VPS13C-depleted cells express lower levels of galectin-12 than galectin-12 knockdown cells yet exhibit lesser adipogenesis suppression is somewhat surprising and probably suggests that VPSC13depletion sets off other pathways with opposing effects on adipocyte differentiation. Genetic studies have found that variants of *VPS13C* are associated with a variety of disorders, including glucose homeostasis [48–54], cancer [55,56], as well as neurodegenerative diseases [42,44]. This again suggests that the protein could have multiple targets and regulate a number of diseases. We envisage that the discovery of this novel pathway of galectin-12 regulation by VPS13C could open up novel avenues for the treatment of metabolic disorders.
